# Salivary IgG Antibody Response to SARS-CoV-2 as a Non-Invasive Assessment of Immune Response—Differences Between Vaccinated Children and Adults

**DOI:** 10.3390/biomedicines14010102

**Published:** 2026-01-03

**Authors:** María Noel Badano, Irene Keitelman, Matías Javier Pereson, Natalia Aloisi, Florencia Sabbione, Patricia Baré

**Affiliations:** 1 Instituto de Medicina Experimental (IMEX)-Consejo Nacional de Investigaciones Científicas y Técnicas (CONICET), Academia Nacional de Medicina, J.A. Pacheco de Melo 3081, Buenos Aires CABA 1425, Argentinapatobare@gmail.com (P.B.); 2 Instituto de Investigaciones Hematológicas (IIHEMA), Academia Nacional de Medicina, J.A. Pacheco de Melo 3081, Buenos Aires CABA 1425, Argentina

**Keywords:** SARS-CoV-2, salivary IgG antibody response, systemic IgG antibody response, vaccines, children

## Abstract

**Background**: Studies comparing systemic and salivary antibody responses against SARS-CoV-2 between children and adults show conflicting results. Furthermore, it is still unclear whether salivary antibody testing could be a non-invasive approach to evaluate the humoral immune response. **Methods**: anti-SARS-CoV-2 IgG antibodies were measured in blood and saliva sample pairs from vaccinated adults to investigate whether salivary antibody response could be a non-invasive assessment of immune response. Salivary antibody levels were also compared between vaccinated children and adults to investigate local antibody responses. **Results**: Salivary IgG antibody response against SARS-CoV-2 largely reflects the systemic response in vaccinated adults. Salivary and systemic antibody concentrations were higher in vaccinated adults who had been infected, received schemes including mRNA-based vaccines, had more exposures, and a shorter time from last exposure. Salivary antibody detection was associated with schemes including mRNA-based vaccines, time from last exposure, and systemic antibody concentrations. Vaccinated children showed higher salivary antibody concentrations than adults. This difference remained when comparing antibody levels between children and adults under equal conditions (vaccination schemes, number of exposures, time from last exposure, COVID-19 history). Younger age, number of exposures, schemes including mRNA-based vaccines, and shorter time from last exposure were associated with salivary antibody levels in a multivariable linear regression analysis (*p* < 0.0001). **Conclusions**: Salivary antibody determination against SARS-CoV-2 could be a non-invasive assessment of the short-term immune response in adults with multiple exposures. Furthermore, the stronger salivary antibody response in children suggests that local immune protection may differ between children and adults, contributing to different outcomes.

## 1. Introduction

While several advances have been made in the understanding of SARS-CoV-2 infection and its associated pathology, certain aspects related to the immune response remain uncertain. Furthermore, the lower availability of blood samples from children coupled with the late administration of COVID-19 vaccines in this population led to less knowledge about their immune response following infection and vaccination in comparison to adults.

It is still debatable whether children and adults show similar systemic humoral immune responses against SARS-CoV-2 after infection and vaccination. Studies have reported similar [1,2], lower [3,4] or stronger and longer-lasting [5,6] systemic antibody responses to SARS-CoV-2 infection in children than adults. There are also conflicting results regarding antibody responses following vaccination, with studies showing higher [6,7], similar [8,9,10] or lower [4] SARS-CoV-2-specific antibody levels in children compared to adults.

Information about the mucosal immune response against SARS-CoV-2 in children is limited. Using saliva-based tests, the prevalence of salivary anti-SARS-CoV-2 IgG antibodies in unvaccinated and vaccinated children has been studied [11,12,13], further showing that salivary antibody levels increase following infection or household contact exposure [12,14] and vaccination [13]. However, studies comparing salivary antibody responses against SARS-CoV-2 between children and adults have yielded contradictory results, showing higher [15] or lower [4] salivary antibody levels in children than in adults.

On the other hand, results from studies comparing systemic and salivary humoral immune responses to SARS-CoV-2 are inconsistent. While several studies have shown a positive correlation between systemic and salivary anti-SARS-CoV-2 IgG antibody levels in both adults [16,17,18] and children [11,15], other studies have shown a weak correlation [19,20]. We have also previously shown that SARS-CoV-2 breakthrough infections in vaccinated adults boost antibody levels not only in the blood but also in the salivary compartment [21]. Therefore, although several studies have demonstrated the validity of assessing specific salivary antibodies as indicators of seroconversion after natural infection or vaccination [16,17,18,22,23], it remains unclear whether saliva could be suitable as a non-invasive alternative to blood for monitoring antibodies against SARS-CoV-2.

In this work we investigated and compared the systemic and salivary IgG antibody responses against SARS-CoV-2 in vaccinated adults, to analyze whether the determination of salivary antibodies against SARS-CoV-2 could be a non-invasive approach to evaluate the humoral immune response following infection or vaccination. Salivary antibody levels were also compared between vaccinated children and adults to analyze whether the specific humoral immune response differed within the salivary compartment.

## 2. Materials and Methods

### 2.1. Study Design, Participants and Samples

Since the beginning of the COVID-19 pandemic, we conduct an observational prospective cohort study to investigate the humoral immune response against SARS-CoV-2 after infection and vaccination. Adult participants were healthcare workers from our institution, the Academia Nacional de Medicina. Child participants were sons and daughters of healthcare workers and their schoolmates. In terms of shared households, 42 children (48%) were sons or daughters of healthcare workers from our institution (*n* = 24). SARS-CoV-2 diagnoses were conducted in adults and children with COVID-19 symptoms and in some household members without symptoms. If a household member tested positive for SARS-CoV-2 and no isolation measures were taken, all other household members were considered exposed (household contacts), except those who were subsequently diagnosed. SARS-CoV-2 infection was confirmed by RT-PCR in nasopharyngeal/oropharyngeal swabs or in saliva samples. Children and adults were monitored and information regarding demographic characteristic (date of birth, sex), vaccination (dates and schemes), COVID-19 history (date and presence of symptoms in positive cases and household contacts) was completed in an online questionnaire by adults and parents.

Blood (*n* = 98) and saliva (*n* = 131) samples from adults who had received two or three vaccine doses collected between December 2021–July 2022 were included. Paired blood and saliva samples were available for 97 participants. Saliva samples (*n* = 88) from children up to 18 years old who had received two or three vaccine doses collected between February-August 2022 were also included. Samples were collected from uninfected adults and children, as well as from individuals who became infected or were household contacts at any stage of vaccination. Blood and saliva samples from seronegative uninfected (PCR-negative) and unvaccinated subjects (*n* = 14) were used to set up the saliva-based SARS-CoV-2 IgG assay (see below). All subjects with confirmed past SARS-CoV-2 infection had mild disease based on the World Health Organization classification [24]. Information regarding demographic characteristic, COVID-19 history, vaccination schemes, number of exposures (through vaccination, infection or household contact exposure) and time between last exposure and sample collection is detailed in Table 1. Vaccines from the same platform were administered on similar dates and with similar interval time between doses. The study was conducted in accordance with the Declaration of Helsinki, and approved by the Ethical Committee of the Academia Nacional de Medicina (T.I.N° 13458/22; 2/22/CEIANM, 2 March 2022). Written informed consent was obtained from adult participants and from parents for children participants.

### 2.2. Sample Collection and Processing

Plasma or serum samples were obtained immediately after centrifugation of the peripheral blood and stored in aliquots at −20 °C until use. For saliva sampling, individuals provided their first saliva of the day by spitting into a tube, ensuring they had not consumed any food or drink, brushed their teeth, or engaged in other activities prior to collection that could introduce variability. Saliva samples were centrifuged at 17,000× *g* for 10 min (4 °C) and the supernatant was stored at −20 °C until use.

### 2.3. SARS-CoV-2 Antibody ELISA

Detection of anti-spike SARS-CoV-2 IgG antibodies in both blood and saliva was performed by ELISA COVIDAR-IgG (Laboratorios Lemos S.R.L, Buenos Aires, Argentina). The assay plates are coated with a purified mixture of the spike protein and the receptor binding domain (RBD) of SARS-CoV-2 from the ancestral Wuhan strain (GenBank: MN908947). Determination of specific antibodies in blood was performed following the manufacturer’s instructions [21], while the assessment of salivary antibodies was performed under the conditions we had previously established [21]. Briefly, all plasma samples were diluted 1:51 with the provided sample diluent, while saliva samples were used neat. For quantitation of antibody titers, a calibration curve was performed by serial dilutions of the supplied standard (400 BAU/mL, reactive human serum adjusted to WHO First International Standard for human immunoglobulin against SARS-CoV-2, NIBSC Code 20/136, version 2.0 of 17 December 2020). Diluted plasma samples, undiluted saliva samples, standard dilutions, and the provided negative control were added to the plate (200 µL). Following an incubation step for 1 h at 37 °C, wells were washed six times and incubated with the conjugate solution (100 µL) for 30 min. at 37 °C. After 6 washes, the substrate solution was added (100 µL) and incubated for 30 min. at 37 °C. The stop solution was then added (100 µL) and sample’s optical densities (OD) were measured immediately at 450 nm. Results were considered reactive when OD 450 nm values were equal to or greater than the cut-off, defined as the OD 450 nm value of the Negative Control plus 0.150 with an additional 10% margin, as specified by the manufacturer. Antibody concentrations were expressed in binding antibody units (BAU) per mL (BAU/mL) and calculated by interpolating the OD 450 nm values of the samples into the calibration curve. Samples yielding OD values exceeding the upper limit of the calibration curve were re-analyzed after appropriate dilution to ensure measurements were obtained within the linear range of the curve.

### 2.4. Performance Characteristics of the Saliva-Based SARS-CoV-2 IgG Antibody ELISA

To assess the diagnostic performance of the saliva-based SARS-CoV-2 IgG antibody ELISA, sensitivity and specificity were calculated using the blood-based ELISA COVIDAR results as the gold standard. Therefore, test results that were positive for SARS-CoV-2 antibodies in both saliva and blood sample pairs were considered true positives; while those that were negative in both sample types were classified as true negatives. Those test results that were positive in blood but negative in saliva were categorized as false negatives. Conversely, test results positive in saliva but negative in blood were defined as false positives.

### 2.5. Statistical Analysis

Data analyses were performed using the GraphPad 10.4.2 Prism software (GraphPad Software, San Diego, CA, USA). Geometric mean concentrations (GMC) of specific antibody levels with 95% confidence intervals (95% CI) were calculated. Antibody levels were analyzed according to variables previously reported to be associated with systemic anti-SARS-CoV-2 IgG antibody levels (age, sex, number of exposures, vaccination schemes, SARS-CoV-2 infection and time between most recent antigen exposure and sample collection). Antibody levels between two groups were compared with Mann–Whitney test. A multivariable linear regression model was performed including these variables as independent variables and salivary antibody levels as the dependent variable. The linear regression coefficients (β) with 95% CI were calculated. The Spearman coefficient of rank correlation was used to assess the correlation between specific salivary and blood antibodies. In all cases, a value of *p* < 0.05 was considered indicative of a significant difference.

## 3. Results

### 3.1. IgG Specific Salivary and Blood Antibody Responses in Vaccinated Adults

**Figure 1:**
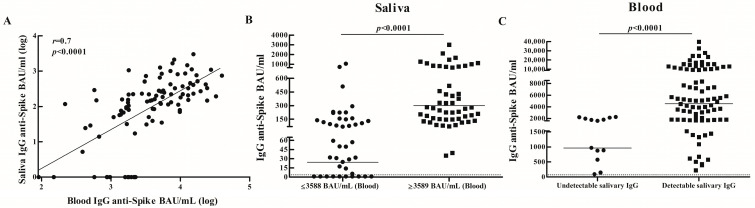
Systemic and salivary antibody responses against SARS-CoV-2 in vaccinated adults. (**A**) Correlation between systemic and salivary SARS-CoV-2-specific IgG responses. Antibody levels were measured in blood and saliva sample pairs (*n* = 97) from vaccinated adults. Correlation between specific salivary and blood antibodies was analyzed by the Spearman rank correlation test. Spearman correlation coefficient (*r*) and *p*-value are indicated. IgG antibody concentrations (BAU/mL) against the SARS-CoV-2 spike protein (Ancestral Wuhan strain) are presented in log. (**B**) Salivary anti-SARS-CoV-2 antibody concentrations were compared between subjects with higher systemic antibody levels (≥GMC: 3589 BAU/mL) and subjects with lower systemic antibody levels (≤GMC: 3588 BAU/mL). (**C**) Comparison of systemic anti-SARS-CoV-2 antibody levels between subjects with or without detectable salivary antibodies. IgG antibody concentrations (BAU/mL) against the SARS-CoV-2 spike protein (Ancestral Wuhan strain) with geometric means are shown. Dotted line indicates the assay detection limit (4.03 BAU/mL). *p* values were determined by Mann–Whitney test (**B**,**C**). Samples were assayed in duplicate and the results shown are representative of one of two independent experiments performed.

Considering total samples, specific antibodies were detected in 100% (*n* = 98) of blood samples (GMC BAU/mL, 95% CI: 3589, 2800–4601) and in 84% (*n* = 131) of saliva samples (GMC BAU/mL, 95% CI: 78.4, 53.9–114.0). Among blood and saliva sample pairs (*n* = 97), antibodies were detected in 87% of saliva samples (Table S1). A positive correlation was observed between levels of specific antibodies in blood and saliva (*r* = 0.7, *p* < 0.0001) (Figure 1A). Subjects with higher systemic antibody levels (≥GMC: 3589 BAU/mL) also showed higher salivary antibody concentrations compared to those with lower systemic antibody levels (≤GMC: 3588 BAU/mL) (GMC BAU/mL, 95% CI, Saliva: 300.3, 227.1–397.2 vs. 24.3, 12.3–48.1; *p* < 0.0001) (Figure 1B). Furthermore, higher systemic antibody levels were found in subjects with detectable salivary antibodies compared to those without detectable salivary antibodies (GMC BAU/mL, 95% CI, Blood: 4529, 3556–5767 vs. 964.8, 515.5–1806; *p* < 0.0001) (Figure 1C).

**Figure 2:**
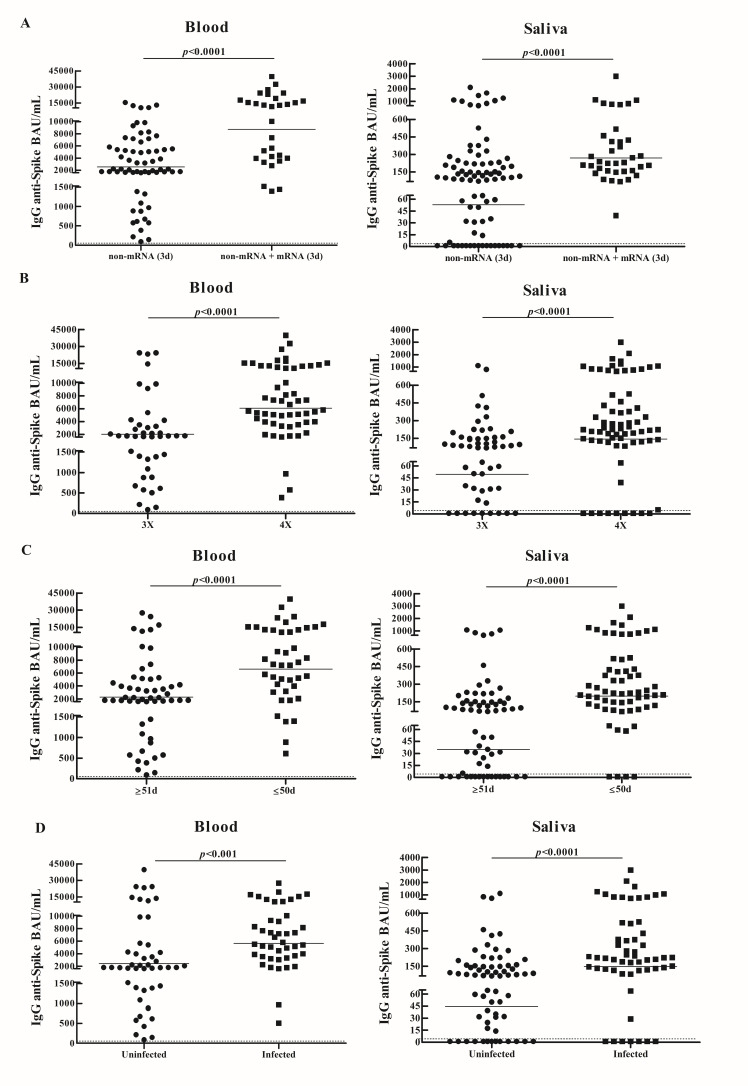
Comparison of systemic and salivary IgG antibody responses against SARS-CoV-2 in vaccinated adults. Blood and salivary anti-SARS-CoV-2 antibody levels were analyzed in vaccinated adults (*n* = 131) according to: vaccination schemes (**A**), number of exposures (**B**), time between last exposure and sample collection (**C**), SARS-CoV-2 infection (**D**). IgG antibody concentrations (BAU/mL) against the SARS-CoV-2 spike protein (Ancestral Wuhan strain) with geometric means are shown. Dotted line indicates the assay detection limit (4.03 BAU/mL). *p* values were determined by Mann–Whitney test. Samples were assayed in duplicate and the results shown are representative of one of two independent experiments performed. non-mRNA (3d), three doses of non-mRNA-based-vaccines; non-mRNA + mRNA (3d), three doses combining mRNA and non-mRNA-based vaccines.

Therefore, we analyzed how variables previously reported to be associated with systemic anti-SARS-CoV-2 IgG antibody levels (see Materials and Methods for details), relate to antibody levels in both the blood and the salivary compartment. Similar results were observed in the blood and in the salivary compartment. The results of the variables where significant differences in antibody levels were observed are shown. As similar results were observed among subjects who received one or two doses of mRNA-based vaccines, they were combined into a single group (non-mRNA + mRNA (3d)) for results presentation. Levels of anti-spike SARS-CoV-2 IgG antibodies were higher in subjects who received three doses of schemes that included mRNA-based vaccines (non-mRNA + mRNA (3d)) compared to those in individuals who received three doses of schemes not including mRNA-based vaccines (non-mRNA (3d)) (GMC BAU/mL, 95% CI, Blood: 8745, 6102–12533 vs. 2574, 1949–3398; *p* < 0.0001; Saliva: 269.0, 199.7–362.4 vs. 53.1, 31.7–88.7; *p* < 0.0001) (Figure 2A). Subjects with a greater number of exposures to SARS-CoV-2 antigens showed higher antibody concentrations than those with a lower number of exposures (GMC BAU/mL, 95% CI, Blood: 6079, 4702–7859 vs. 2033, 1384–2988; *p* < 0.0001; Saliva: 142.7, 84.7–240.4 vs. 49.5, 28.9–84.5; *p* < 0.0001) (Figure 2B). Antibody concentrations decreased as the interval time between last exposure and sample collection increased (GMC BAU/mL, 95% CI, Blood: 6574, 4840–8929 vs. 2326, 1673–3234; *p* < 0.0001; Saliva: 198.9, 129.6–305.2 vs. 34.9, 19.9–61.2; *p* < 0.0001) (Figure 2C). Adults who had been infected with SARS-CoV-2 showed higher antibody levels than uninfected subjects (GMC BAU/mL, 95% CI, Blood: 5660, 4379–7317 vs. 2410, 1636–3549; *p* < 0.001; Saliva: 147.8, 84.1–259.8 vs. 44.7, 27.0–73.9; *p* < 0.0001) (Figure 2D).

Using Fisher’s exact test, detection of specific antibodies in saliva was associated with schemes including mRNA-based vaccines (*p* < 0.001; OR = 21.5), the time between last exposure and sample collection (*p* < 0.01; OR = 4.7) and systemic antibody concentrations (*p* < 0.0001; OR = 43.6).

By using the antibody results from blood and saliva sample pairs (*n* = 97), diagnostic performance for the salivary assays was calculated considering the serum ELISA results as the reference standard. The saliva-based SARS-CoV-2 IgG antibody ELISA test showed a sensitivity of 87% (95% CI: 78.2–92.7) and a specificity of 100% (95% CI: 76.8–100) for IgG detection.

### 3.2. Comparison of IgG Specific Salivary Antibody Responses Between Vaccinated Children and Adults

**Figure 3:**
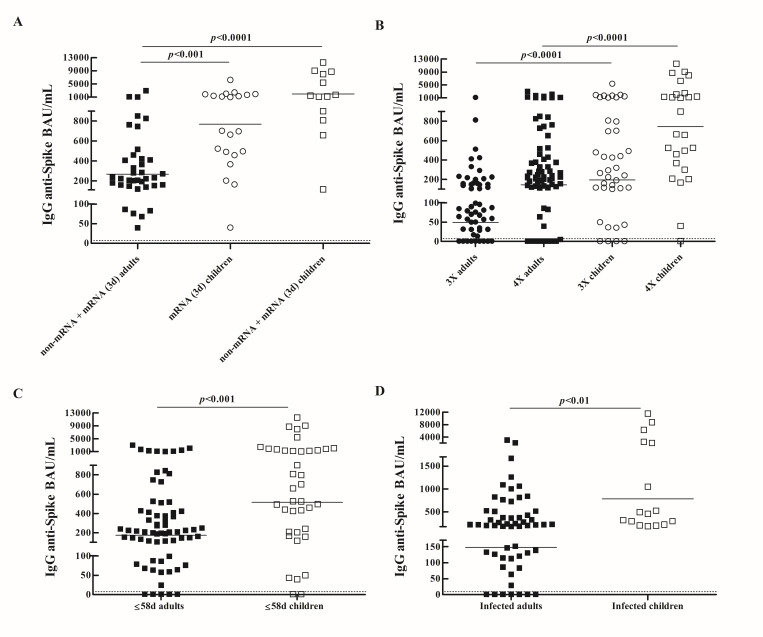
Comparison of salivary antibody responses against SARS-CoV-2 between vaccinated children and adults. Salivary anti-SARS-CoV-2 antibody levels were compared between vaccinated adults (*n* = 131) and children (*n* = 88) according to: vaccination schemes (**A**), number of exposures (**B**), time between last exposure and sample collection (**C**), SARS-CoV-2 infection (**D**). IgG antibody concentrations (BAU/mL) against the SARS-CoV-2 spike protein (Ancestral Wuhan strain) with geometric means are shown. Dotted line indicates the assay detection limit (4.03 BAU/mL). *p* values were determined by Mann–Whitney test. Samples were assayed in duplicate and the results shown are representative of one of two independent experiments performed. non-mRNA + mRNA (3d), three doses combining mRNA and non-mRNA-based vaccines; mRNA (3d), three doses of mRNA-based vaccines.

Considering all samples, children showed higher salivary antibody concentrations compared to adults (GMC: 157.1 BAU/mL, 95% CI: 91.9–268.3 vs. 79.8 BAU/mL, 95% CI: 54.9–115.9; *p* < 0.01). Therefore, antibody levels were compared between adults and children who shared the same conditions (vaccination schemes, number of exposures, time from last exposure, COVID-19 history). Children who received three doses of mRNA vaccines (mRNA (3d)) (GMC: 769.3 BAU/mL; 95% CI: 466.6–1268) or three doses combining mRNA and non-mRNA-based vaccines (non-mRNA + mRNA (3d)) (GMC: 1987 BAU/mL; 95% CI: 878.7–4492) showed higher antibody concentrations than adults who received three doses of schemes including mRNA-based vaccines (*p* < 0.001) (Figure 3A). Salivary antibody titers were higher in children compared to adults who had the same number of exposures (GMC BAU/mL, 95% CI, 3X: 195.0, 96.6–393.7 vs. 49.5, 28.9–84.5; *p* < 0.0001; 4X: 744.6, 340.8–1627 vs. 142.7, 84.7–240.4; *p* < 0.0001) (Figure 3B). Antibody concentrations were higher in children compared to adults who had the same interval time (≤58 days) between last exposure and saliva collection (GMC: 515.2 BAU/mL, 95% CI: 267.1–993.9 vs. GMC: 174.0 BAU/mL, 95% CI: 113.1–267.8; *p* < 0.001) (Figure 3C). Children who had been infected with SARS-CoV-2 showed higher antibody levels than adults who had been infected (GMC BAU/mL, 95% CI: 783.2, 368.1–1667 vs. 147.8, 84.1–259.8) (*p* < 0.01) (Figure 3D). Multivariable linear regression analysis showed that younger age (β1: 874.2, 95% CI: 468.6–1280; *p* < 0.0001), number of exposures (β2: 364.0, 95% CI: 3.6–724.3; *p* < 0.05), schemes including mRNA-based vaccines (β3: 560.6, 95% CI: 147.4–973.7; *p* < 0.01), and shorter time from last exposure (β4: 373.6, 95% CI: 3.5–748; *p* < 0.05) were associated with salivary antibody levels (*p* < 0.0001).

## 4. Discussion

Data from studies comparing antibody responses against SARS-CoV-2 after infection or vaccination between children and adults are conflicting in blood [1,2,3,4,5,6,7,8,9,10] and in the mucosa [4,15]. On the other hand, as studies comparing systemic and salivary humoral immune responses to SARS-CoV-2 have yielded contradictory results [11,15,16,17,18,19,20,21,22], it remains yet to be determined whether saliva could be used as a non-invasive alternative to blood for the determination of antibodies against SARS-CoV-2.

In this work, we investigated and compared the systemic and salivary IgG antibody responses against SARS-CoV-2 in vaccinated adults. Analysis of different variables previously reported to be related to systemic anti-SARS-CoV-2 IgG antibody levels showed similar results for blood and saliva. Higher antibody concentrations were observed in both the blood and saliva of vaccinated adults who had been infected with SARS-CoV-2, received schemes including mRNA-based vaccines, had more exposures, and a shorter interval time between last exposure and sample collection. Furthermore, a positive correlation was found between systemic and salivary anti-SARS-CoV-2 IgG antibody levels, with higher salivary antibody concentrations observed in subjects with higher systemic antibody levels. Overall, specific antibodies were detected in 100% of blood samples and in 84% of saliva samples; while among paired blood–saliva samples antibodies were detected in 87% of saliva samples. Detection of specific salivary antibodies was associated with schemes that included mRNA-based vaccines, time between last exposure and sample collection, and systemic antibody concentrations. These results, together with the fact that most subjects without detectable salivary antibodies showed lower systemic antibody levels, suggest that salivary antibody detection could depend on variables that influence systemic antibody levels. Therefore, although our results showed that the salivary immune response against SARS-CoV-2 in vaccinated adults largely reflects that observed at systemic levels, salivary antibody detection might not be possible in subjects presenting lower systemic antibody concentrations. This is in agreement with previous results showing that salivary antibody responses were mainly detected in subjects showing higher serum antibody levels [25].

As far as we are concerned, it seems that differences among previous studies likely reflect variations in the cohorts analyzed. Studies of unvaccinated, infected individuals have generally reported lower salivary antibody prevalence [11,13,25], whereas higher detection rates have been observed in vaccinated populations [12,13,18,22,23]. In our study, the high prevalence of salivary antibodies is likely due to multiple SARS-CoV-2 exposures in most participants, leading to higher systemic antibody levels and improved salivary detection. In addition, salivary antibody detection was associated with time since last exposure. Together, these findings suggest that saliva-based assays are more effective in individuals with multiple and recent exposures through infection or vaccination. This may explain the low detection rates reported early in the pandemic, when vaccination coverage was limited [11,13,25]. Given the current endemic circulation of SARS-CoV-2 and high vaccination coverage, saliva-based antibody testing is likely to be more suitable for antibody measurement. In this context, our study aims to contribute additional evidence using a standardized and internally consistent methodological approach, including paired saliva and blood samples analyzed with the same validated ELISA platform and quantified using WHO-traceable binding antibody units (BAU/mL).

The performance characteristics of the saliva-based SARS-CoV-2 IgG antibody ELISA were calculated using the serum ELISA results as the gold standard. Although the test demonstrated adequate specificity, its sensitivity was slightly below the threshold generally considered necessary for diagnostic use (≥90%). Nevertheless, applying the test to selected populations (subjects with multiple and recent antigen exposures) may improve sensitivity to recommended levels. The performance of the saliva-based SARS-CoV-2 IgG antibody ELISA could be further improved through methodological optimization, including minor technical modifications and increased sample size.

We also compared salivary antibody concentrations between vaccinated children and adults to analyze whether specific antibody responses differed within the salivary compartment. Vaccinated children showed a higher salivary antibody response against SARS-CoV-2 compared to adults. This difference remained when antibody levels were compared between vaccinated children and adults who shared the same conditions (vaccination schemes, number of exposures, time from last exposure, COVID-19 history). Multivariable linear regression analysis showed that younger age, number of exposures, schemes including mRNA-based vaccines, and shorter time from last exposure were associated with salivary anti-SARS-CoV-2 IgG antibody levels.

The biological and molecular basis for a favorable outcome after SARS-CoV-2 infection in children compared to adults is not fully understood. Several hypotheses have tried to explain the milder presentation of the disease in children, including a protective role of pre-existing cross-reactive antibodies against seasonal coronaviruses, a lower expression of the angiotensin-converting enzyme 2 (ACE2) and a less propensity to develop an exacerbated pro-inflammatory response, among others [26]. However, the contribution of the antibody response against SARS-CoV-2 to the favorable outcome in the pediatric population is not entirely clear. In the current work, we showed that children had higher salivary anti-SARS-CoV-2 IgG antibody concentrations than adults. Given that mucosal immunity plays a key role in prevention and early defense against infection, being the entry route for the virus and the site of first encounter with the immune response, it is tempting to speculate that the stronger salivary antibody response observed in children could help prevent or limit infection, thus contributing to the favorable outcome observed in the pediatric population.

While children generally developed asymptomatic infection or mild COVID-19, other respiratory viruses, such as the respiratory syncytial virus (RSV) and influenza can cause severe respiratory illness in young children. Direct comparisons of salivary versus serum IgG responses between children and adults under comparable vaccination or exposure conditions remain limited for influenza and RSV [27,28]. Therefore, a more complete understanding of age-related effects on local and systemic immune responses to respiratory viruses and their relationship with clinical presentation is warranted.

Several studies have compared systemic antibody responses between children and adults after infection and vaccination. However, although some studies have compared salivary antibody responses between these populations, the majority have focused on unvaccinated infected children and adults [14,15]. Only a limited number of studies have examined salivary antibody responses in vaccinated children, and these have not included a comparison with adults [11,12,13]. To the best of our knowledge, only one study has compared salivary IgG antibody levels between vaccinated children and adults, showing higher salivary antibody levels in adults [4]. However, in the study of Padoan et al., saliva samples were collected using Salivette and salivary and blood antibody levels were measured using different techniques (ELISA vs. CLIA) [4]. Instead, in our study, saliva was obtained by passive flow and paired saliva and blood samples were analyzed with the same validated ELISA platform. In addition, the lack of detailed information regarding vaccination schemes in that study, such as the number of doses and vaccine brands administered in both children and adults, limits direct comparisons and precludes definitive conclusions. Therefore, methodological differences, including saliva collection procedures, techniques used to measure antibodies, the absence of harmonized international controls, and the antigenic target used in saliva-based assays, among others, could underlie the contradictory findings in the literature. Further studies are needed to clarify age-related differences in salivary antibody responses among vaccinated populations.

While the systemic and mucosal compartments of the immune system are distinct and largely independent, an interrelationship often occurs [29,30]. Nevertheless, in the context of SARS-CoV-2 infection, the interplay between systemic and mucosal antibody responses, as well as the extent to which they influence one another, remains incompletely understood. Salivary IgG is primarily derived from circulating IgG through transudation [31]. In our study, we observed that the salivary IgG response in adults highly reflects the systemic antibody response and a positive correlation between levels of specific antibodies in blood and saliva. As other authors have pointed out, this strong correlation between serum and saliva suggests that salivary antibodies are derived primarily from transudation from the blood rather than local production [23]. However, in children, it remains to be determined whether the salivary antibody response also similarly reflects that observed in blood. In addition, given the possibility of some local IgG production, a difference in the locally mounted antibody response may also contribute to the observed difference in the salivary antibody response against SARS-CoV-2 between children and adults. Further studies are warranted to elucidate the interplay and functional efficiency of systemic and mucosal immune compartments, and to determine whether these interactions contribute to age-related differences in local antibody responses.

Despite comparable vaccination schemes, number of exposures, time since last exposure, and COVID-19 history, children in our cohort exhibited higher salivary anti–SARS-CoV-2 IgG concentrations than adults. This difference persisted after adjustment for these variables in multivariable analyses, suggesting that age itself contributes independently to the magnitude of the salivary antibody response. Although our study was not designed to investigate underlying mechanisms, several age-related factors may help explain this observation. Children display a distinct mucosal immune environment in the upper respiratory tract, characterized by heightened baseline immune activity and more recent antigenic stimulation, which may favor stronger local antibody responses [32]. In contrast, age-associated immune regulation and immunosenescence in adults may contribute to lower or more rapidly waning mucosal IgG levels [33]. These hypotheses provide biological context for our findings but require dedicated mechanistic studies to be directly tested.

Limitations of this study included the lack of information on the systemic antibody response in children, which precluded the comparison between systemic and salivary immune responses in them. The limited availability of saliva samples (especially those from children that were diluted several times), prevented further investigation on neutralizing and specific IgA antibody responses. However, some reports showed that salivary anti-SARS-CoV-2 IgG antibody levels strongly correlate with the neutralizing capacities of both serum [18] and saliva [23]. Given that IgA is the key immunoglobulin involved in mucosal immunity, and considering the inconsistent evidence regarding SARS-CoV-2-specific salivary IgA and neutralizing antibody responses, further studies investigating these responses could also add insights related to the local immune protection in children and adults. Information about SARS-CoV-2 variants involved in the infections/household contact exposures and their association with antibody levels was not available. However, most infections/household contact exposures, both in adults and children, occurred when the only circulating variant in Argentina was Omicron. Taking this into account, it would be interesting to investigate salivary IgG responses against Omicron antigens to obtain information related to mucosal immune protection against new emerging viral variants. These limitations highlight the need for further research to better understand salivary and systemic immune responses, especially in the most vulnerable populations, such as young children, the elderly and immunocompromised individuals.

## 5. Conclusions

Altogether, our results showed that the salivary IgG antibody response against SARS-CoV-2 largely reflects the systemic response in vaccinated adults. However, salivary antibodies may not be detectable in subjects with low systemic antibody levels. Nevertheless, since saliva sampling is non-invasive and allows self-collection, salivary antibody determination could be performed as an initial screening to evaluate the antibody response against SARS-CoV-2. In addition, as salivary antibody response is indicative of the systemic, salivary antibody determination could guide vaccination strategies by administering booster doses to subjects without detectable salivary antibodies, allowing them to achieve high systemic antibody levels.

In conclusion, the determination of salivary antibodies against SARS-CoV-2 could be a non-invasive approach for the evaluation of the short-term immune response in subjects with multiple exposures, either resulting from vaccination combined with infection or from the administration of immunogenic vaccination schemes, both in the adult and pediatric population, especially in settings where blood sampling cannot be performed.

Furthermore, the higher levels of salivary anti-SARS-CoV-2 IgG antibodies observed in children suggest that local immune protection may differ between children and adults. Whether the stronger salivary antibody response in children provides improved local immune protection and contributes to the favorable outcome in the pediatric population warrants further investigation.

## Figures and Tables

**Table 1:** Characteristics of children and adults.

	Children (*n* = 88)	Adults (*n* = 131)
**General characteristics**		
Age, median (range), y	10 (4–17)	45 (27–83)
Sex		
Female, No. (%)	40/88 (45)	91/131 (69)
Male, No. (%)	48/88 (55)	40/131 (31)
**COVID-19 history**		
Uninfected, No. (%)	35/88 (40)	67/131 (51)
Confirmed past SARS-CoV-2 infection, No. (%)	16/88 (18)	56/131 (43)
Household contacts, No. (%)	37/88 (42)	8/131 (6)
Infected/household contacts with COVID-19 compatible symptoms, No. (%)	32/53 (60)	55/64 (86)
Infected/household contacts exposed to pre-Omicron variants	15/53 (28)	17/64 (27)
Infected/household contacts exposed to Omicron variant	38/53 (72)	47/64 (73)
**Vaccination schemes non-mRNA-based**		
BBIBP-CorV × 2, No. (%)	46/88 (52)	5/131(4)
ChAdOx1 nCoV-19 × 2, No. (%)		5/131(4)
Sputnik V × 2, No. (%)		1/131(1)
BBIBP-CorV × 2 + ChAdOx1 nCoV-19 × 1, No. (%)	−	66/131 (50)
Sputnik V × 2 + ChAdOx1 nCoV-19 × 1, No. (%)	−	10/131 (8)
ChAdOx1 nCoV-19 × 3, No. (%)	−	7/131 (5)
**Vaccination schemes mRNA-based**		
BNT162b2 mRNA × 2, No. (%)	7/88 (8)	−
mRNA-1273 × 2, No. (%)	1/88 (1)	−
BNT162b2 mRNA × 3, No. (%)	17/88 (19)	−
BNT162b2 mRNA × 2 + mRNA-1273 × 1, No. (%)	4/88 (5)	−
BBIBP-CorV × 2 + BNT162b2 mRNA x1, No. (%)	11/88 (13)	8/131 (6)
BBIBP-CorV × 2 + mRNA-1273 × 1, No. (%)	2/88 (2)	4/131 (3)
Sputnik V × 2 + BNT162b2 mRNA x1, No. (%)	−	8/131 (6)
ChAdOx1 nCoV-19 × 2 + BNT162b2 mRNA x1, No. (%)	−	4/131 (3)
Sputnik V × 1 + mRNA-1273 × 2, No. (%)	−	13/131 (10)
**Number of antigen exposures**		
Two vaccine doses (2X), No. (%)	23/88 (26)	8/131 (6)
Two vaccine doses plus one infection/household contact exposure (3X), No. (%)	27/88 (31)	3/131 (2)
Three vaccine doses (3X), No. (%)	12/88 (14)	59/131 (45)
Two vaccine doses plus two household contact exposures (4X), No. (%)	4/88 (4)	−
Three vaccine doses plus one infection/household contact exposures (4X), No. (%)	22/88 (25)	61/131 (47)
Time from last antigen exposure and sample collection, median (range), days	76 (21–270)	50 (21–234)
Time from last antigen exposure and sample collection, median (range), days	58 (21–270)	

BBIBP-CorV (Sinopharm); ChAdOx1 nCoV-19 (University of Oxford/AstraZeneca); Sputnik V (Gamaleya NRCEM); BNT162b2 mRNA (Pfizer–BioNTech); mRNA-1273 (Moderna).

## Data Availability

The raw data supporting the conclusions of this article will be made available by the authors on request.

## Supplementary Materials

The following supporting information can be downloaded at: https://www.mdpi.com/article/10.3390/biomedicines14010102/s1, Table S1: Antibody levels in paired blood and saliva samples (*n* = 97).

## Abbreviations

BAU	binding antibody units
BAU/mL	binding antibody units per mL
GMC	geometric mean concentrations
95% CI	95% confidence intervals
